# A Taste of Our Own Medicine: Fostering Empathy in Medical Learners Through Patient Simulation

**DOI:** 10.5811/westjem.48535

**Published:** 2025-11-26

**Authors:** Romy Portieles Peña, William Weber

**Affiliations:** * The University of Chicago Medical Center, Department of Internal Medicine, Chicago, Illinois; † Rush University Medical Center, Department of Emergency Medicine, Chicago, Illinois

## Abstract

**Introduction:**

Residents and medical students spend thousands of hours of medical education learning the physician’s perspective but rarely find themselves on the other side of the stethoscope. In this study we evaluated whether a brief, novel curriculum of simulating the patient experience could improve medical learners’ reported empathy for patients and ability to explain medical interventions.

**Curricular Design:**

Fifty-eight medical learners (medical students and resident physicians) participated in a 50-minute didactic session where learners simulated patient experiences such as wearing a patient gown and cervical collar, walking with crutches, and tasting potassium chloride and thickened water. Learners evaluated their perceptions of the curriculum with a survey.

**Impact/Effectiveness:**

Participants reported limited experience as patients, with 66.7% never having been hospitalized and 50% not taking any daily medications. Learners rated the curriculum highly on a seven-point Likert scale with 98% expressing it helped them to empathize with patients (90% either agreed or strongly agreed) and 95% expressing that it would help them explain interventions (81% either agreed or strongly agreed). There was no difference between medical students and residents regarding reported effect on empathy (M 6.24 vs 6.44; *P* = .30) or effect on ability to explain the intervention (M 6.06 vs 6.24; *P* = .43). This brief curriculum simulating the patient experience was well-received by medical student and resident learners, who overwhelmingly felt it improved their empathy for patients and explanations of common interventions. This approach to fostering empathy could help both medical student and resident learners, many of whom may have limited experience as a patient.

## BACKGROUND

Resident physicians and medical students receive extensive medical education focused on thinking like a physician. While trainees have significant clinical knowledge, many lack significant personal experience as a patient, with < 5% of those aged 18–24 years of age being hospitalized per year.^1^ Curricular activities where learners simulate the patient perspective can potentially overcome trainees’ gaps in patient experience. However, such studies have been limited. Some prior studies have maintained a very narrow focus, such as tasting various oral antibiotics or participating in a diabetic shopping experience. 2,3 While such activities can lead to increased empathy, the effects may not easily translate to other domains.

Other studies have involved longer interventions such as an overnight hospital stay or three-hour visit to the emergency department (ED). 4,5 While these broaden the spectrum of experiences encountered, they are very resource intensive for learners and may tax hospitals with limited bed capacity and education funding. A recent simulation-based study of residents role-playing as patients was rated favorably but without measurable improvements in empathy. 6 Empathy has been associated with increased patient satisfaction, patient adherence to plans, and improved clinical outcomes.^7^ We hypothesized that having learners undergo simulated patient interventions would improve reported learner empathy.

## CURRICULAR DESIGN

In this study we piloted a brief patient simulation curriculum employing common but uncomfortable activities that exemplify a spectrum of medical experiences faced by patients. The curriculum was designed using Kern’s six-step approach to curriculum development, with needs assessment from a group of five medical educators and 12 learners. 8 This needs assessment noted a lack of direct experience with the interventions that trainees were learning. Experiences were chosen to cover a broad variety of common medical interventions while also retaining a brevity that allowed for easy integration into existing didactics. We considered but did not pursue other interventions, such as tasting oral antibiotics (risk of side effects) or trying bilevel positive airway pressure (resource intensive).

We hypothesized that this curriculum would increase medical learners’ reported empathy for their patients (primary outcome) and their perceived ability to explain these medical interventions (secondary outcome). This study consisted of a 50-minute didactic session where medical learners simulated patient experiences. Emergency medicine (EM) residents, internal medicine residents, and medical students were recruited to the study. A total of 58 learners participated: 33 medical students and 25 EM or internal medicine residents over the course of two separate days. This study was approved by the University of Chicago Institutional Review Board [IRB21-1203].

During the didactic session, learners were separated into two groups of 12–15 learners, each with an instructor. They followed an activity flow starting with donning patient gowns and taping intravenous tubing to their arms to simulate hospital garb (five minutes). Learners subsequently underwent a “trauma” station where they were fitted with cervical collars and then placed on a hard, trauma backboard with a simplified trauma roll performed by other learners (seven minutes). After the trauma roll, they were instructed to ambulate using crutches (five minutes). Finally, learners experienced a “per os station” where they were given 20 mL of thickened water and 20 mL of a typical potassium chloride oral solution to simulate dysphagia diet and potassium repletion, respectively (five minutes). As trainees transitioned between activities, instructors elicited learner experience and had a 2–3 minute debrief of each activity.

After completing all activities, learners filled out an anonymous survey regarding their perceptions of the curriculum and prior patient experience. Learners used a Likert scale to rate how they felt the study changed their empathy for and explanations to patients. Finally, the survey collected qualitative data focusing on learners’ feelings during their time as “patients” and how the activity might impact their medical practice. After completing the survey, the trainees had a large-group debrief for approximately 10 minutes where they shared their experience and personal learning points. Learners were compensated with a $10 gift card for their participation.

We analyzed survey findings in Stata (StataCorp LLC, College Station, TX) and Microsoft Excel (Microsoft Corporation, Redmond, WA) using two-sample *t*-tests with Bonferroni correction. Qualitative data were coded using an inductive approach to generate themes with two coders. Discrepancies were discussed until coders agreed. A priori power analysis indicated that a sample of 32 participants would provide 80% power to detect a change of 20% in perceived empathy (Cohen *d* = 0.8, α = 0.05), which was chosen as a de novo threshold. This study exceeded that sample size.

## IMPACT / EFFECTIVENESS

A total of 58 learners participated in two separate sessions; 33 medical students and 25 residents, with equal male/female ratio, and all participants completed the survey. Participants reported limited experience as patients, with the majority never having been hospitalized and half taking no daily medications (Table 1).

Learners rated the curriculum highly on a seven-point Likert scale: 99% of participants expressed that the curriculum helped them to empathize with patients, with 90% either agreeing or strongly agreeing (Table 2); and 95% of learners reported that the session would help them better explain interventions to patients, with 81% either agreeing or strongly agreeing. There was no difference between medical students and residents regarding reported effect on empathy (M 6.24 vs 6.44; *P* = .30) or ability to explain interventions (M 6.06 vs 6.24; *P* = .43). However, learners who had never been hospitalized prior to the study had a significantly higher reported increase in empathy compared to learners who had been previously hospitalized (M 6.47 vs 6.05; *P* = .03). There was no difference in reported improvement in explanations to patients between learners who had been hospitalized and those who had not (M 5.85 vs 6.29; *P* = .06). Of those who participated in the session, 97% reported they would change how they would describe interventions to patients based on their experience in the study.

Qualitatively, the two most common themes identified were 1) discomfort leading to reconsideration of interventions; and 2) empathy toward what the patients were experiencing (Table 3). A representative quote of these two changes was as follows: “[The study] will help me prepare patients for uncomfortable parts of their hospitalization and be conscious about when I can back off on uncomfortable interventions.”

## LESSONS LEARNED

We devised a learner handout with the station flow and instructions, which helped learners track their progress. Speech/swallow staff generously provided liquid thickening mix, and hospital pharmacists provided potassium chloride. Learners had the option to change into gowns in the bathroom or don gowns over their clothes. None chose to fully change, which facilitated a discussion about patient vulnerability. When learners had emotional responses to the stimuli, it helped to empathize and then remind them of the shift in magnitude as a patient: “Now imagine you have to drink thickened water every single day from now on.” The one-hour duration was feasible to implement during weekly didactics, and the various stations could support small-group rotations with floating instructors.

This was a single-center study focused on perceived changes in empathy. This study only used a post-survey, which could have led to response shift or recall bias. Future iterations could evaluate higher levels on the Kirkpatrick model to establish improved communication or change in practice. The questions of the validated Jefferson Scale of Empathy had a focus beyond the scope of this intervention but could be considered as a future measure.

## Figures and Tables

**Table 1:** Baseline demographic information of medical student/resident learners who participated in a didactic session that simulated patient experience.

Learner demographics	Count (percentage)
Training level	
Medical student	33 (57%)
Resident	25 (43%)
Sex	
Female	29 (50%)
Male	29 (50%)
Prior hospitalizations	
Never	38 (66%)
Once	15 (26%)
Twice	2 (3%)
≥ Three times	3 (5%)
Daily medication use	
Yes	29 (50%)
No	29 (50%)

**Table 2:** Survey findings of medical student/resident learners who participated in a didactic session that simulated patient experience.

This activity:	Strongly disagree	Disagree	Slightly disagree	Neutral	Slightly agree	Agree	Strongly agree
Helps me empathize with patients	0	0	0	1 (2%)	5 (9%)	26 (45%)	26 (45%)
Helps me explain interventions to patients	0	0	0	3 (5%)	8 (14%)	25 (43%)	22 (38%)

**Table 3:** Qualitative themes in survey of participants in a didactic session that simulated patient experience.

Themes identified	Example	Percentage of learners noting theme
Discomfort with interventions	“The C-collar is really uncomfortable.”	57%
Empathy toward patients	“Opened my eyes to the challenges of being a patient”	24%
Vulnerability	“It made me feel vulnerable and uncomfortable.”	10%
Gratitude	“I feel appreciative of my health.”	5%
